# PSMA-Targeted Nanotheranostics for Imaging and Radiotherapy of Prostate Cancer

**DOI:** 10.3390/ph16020315

**Published:** 2023-02-17

**Authors:** Niranjan Meher, Henry F. VanBrocklin, David M. Wilson, Robert R. Flavell

**Affiliations:** 1 Department of Radiology and Biomedical Imaging, University of California, San Francisco, CA 94143, USA; 2 Helen Diller Family Comprehensive Cancer Center, University of California San Francisco, San Francisco, CA 94143, USA; 3 Department of Pharmaceutical Chemistry, University of California, San Francisco, CA 94158, USA

**Keywords:** prostate-specific membrane antigen (PSMA), nanocarriers, nanoparticle, enhanced permeability and retention (EPR) effect, small-molecule PSMA inhibitors, prostate cancer theranostics, radioligand theranostics, radiopharmaceutical therapy, positron emission tomography (PET)

## Abstract

Targeted nanotheranostic systems offer significant benefits due to the integration of diagnostic and therapeutic functionality, promoting personalized medicine. In recent years, prostate-specific membrane antigen (PSMA) has emerged as an ideal theranostic target, fueling multiple new drug approvals and changing the standard of care in prostate cancer (PCa). PSMA-targeted nanosystems such as self-assembled nanoparticles (NPs), liposomal structures, water-soluble polymers, dendrimers, and other macromolecules are under development for PCa theranostics due to their multifunctional sensing and therapeutic capabilities. Herein, we discuss the significance and up-to-date development of “PSMA-targeted nanocarrier systems for radioligand imaging and therapy of PCa”. The review also highlights critical parameters for designing nanostructured radiopharmaceuticals for PCa, including radionuclides and their chelators, PSMA-targeting ligands, and the EPR effect. Finally, prospects and potential for clinical translation is discussed.

## 1. Introduction

Targeted Nanocarrier platforms hold significant promise to deliver target-specific pharmaceuticals for the imaging and therapy of cancer [1,2]. Despite high targeting affinity and tumor penetration, most low-molecular-weight drugs suffer from rapid clearance, even from the tumor, potentially requiring high and/or multiple dose treatment to achieve optimum therapeutic efficacy, with associated off-target toxicity [3]. Nanocarrier systems, such as inorganic nanoparticles (NPs), organic supramolecular self-assemblies, liposomes, and macromolecules, have the potential to improve the solubility and stability of encapsulated drug molecules with prolonged blood circulation for better therapeutic efficacy [1]. Along with the delivery of high payloads, nanocarrier scaffolds enable encapsulation of multiple contrast agents and therapeutic drugs. Their large surface area allows multifunctionality by conjugating a diversity of targeting ligands, including small molecules, carbohydrates, aptamers, nucleic acid peptides, and antibodies, to improve targeted delivery [1,2]. The extravasation of nanocarriers into tumors depends on how effectively they can escape from the reticuloendothelial system cells in the spleen and liver. By optimizing particle size, the hydrophilic polymer coating of nanocarriers can improve tumor retention by avoiding fast renal clearance. However, as the size of the nanocarriers increases beyond 12 nm, the EPR-mediated passive uptake tends to overshadow the target-specific tumor uptake and retention depending on tumor phenotypes [4]. In addition, most prostate cancers (PCa) have less permeable vasculature resulting in low non-penetrating EPR-mediated tumor accumulation of the large-size nanocarriers [5,6,7]. Thus, careful selection of size is a crucial part of developing targeted nanocarriers for PCa theranostics.

Glutamate carboxypeptidase II (GCPII), also known as prostate-specific membrane antigen (PSMA) is a well-validated target for imaging and treatment of PCa, as it is overexpressed in most primary PCa lesions along with distant metastatic lesions and metastatic lymph nodes [8]. The GCPII is a class II transmembrane glycoprotein that hydrolyses one of the highly prevalent neurotransmitters, N-Acetyl-l-aspartyl-l-glutamate (NAAG) [9]. This peptidase is expressed in several different tissues, such as the salivary glands, intestines, kidneys, and brain, but is upregulated in PCa by 100–1000-fold and serves as a potent target for PCa theranostics. GCPII was first cloned from human prostate parenchyma and designated as prostate-specific membrane antigen (PSMA) [9,10]. The report of the PSMA crystal structure in 2005 provided an understanding of the structure and conformation of the active pocket of PSMA and facilitated the rational design of highly efficient small molecule-based PSMA inhibitors [11]. Low-molecular-weight ligands, especially urea-glutamate derivatives, possess many advantages including high PSMA binding, biological stability, and easy large-scale synthesis, and have generally received preference over antibodies and aptamers [12]. A variety of other surface targets are currently under evaluation in prostate cancer, including bombesin [13], prostate stem cell antigen (PSCA) [14], CUB-domain-containing protein 1 (CDCP-1), CD46 [15,16], human kallikrein 2 (hK2) [17], delta-like ligand 3 (DLL3) [18,19,20], and TROP-2 [21]. Nevertheless, due to the major advantages of high tumor and low background expression, and highly optimized targeting ligands, PSMA remains by far the most thoroughly investigated theranostic target in prostate cancer [22,23].

**Figure 1:**
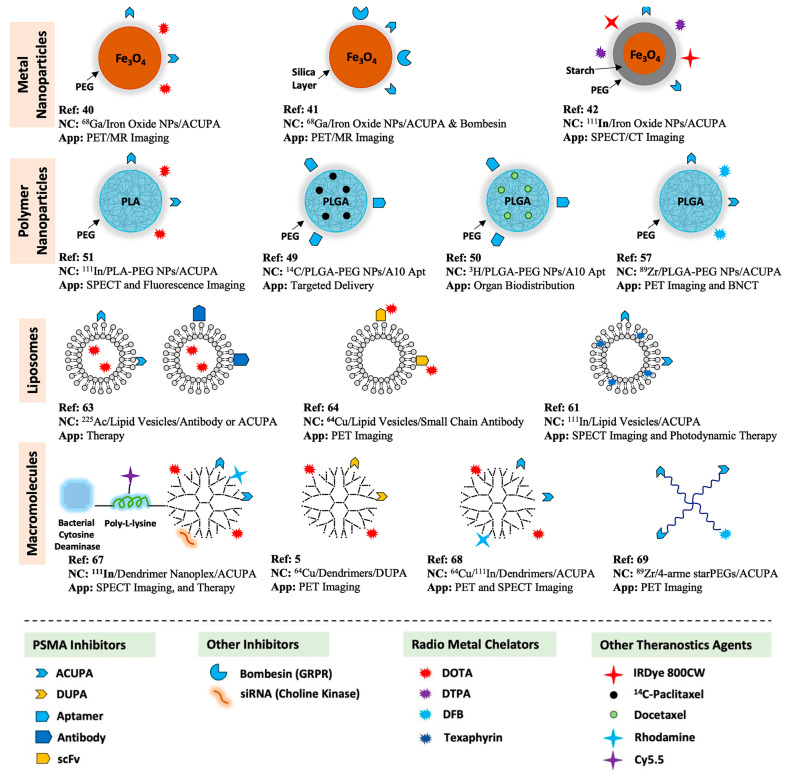
Summary of PSMA-targeted nanocarriers for radioligand imaging and treatment of PCa.

PSMA-targeted radionuclide theranostics have been intensely studied over the last several years, resulting in the FDA approval of [^68^Ga]PSMA-11 and [^18^F]DCFPyL for PET imaging, and [^177^Lu]PSMA-617 for radiopharmaceutical therapy of PCa [8,24,25,26,27]. Over the years, various attempts have been made to integrate radioligand imaging and therapy with PSMA-targeted nanosystems designed to improve PCa therapeutic efficacy. Many features may influence the performance of target-specific nanocarriers, as several parameters such as size, surface charge, targeting ligand density, and other parameters potentially influence their in vivo pharmacokinetics [1]. In this review, we summarize the up-to-date development of PSMA-targeted nanocarriers as PCa theranostics, including hydrophilic polymers, supramolecular NPs, liposomes, metal NPs, and dendrimers (Figure 1). Literature searches including the terms “PSMA-targeted + Nanocarrier/Nanoparticle/Nanomedicine/Macromolecule/Polymer + Radioligand Imaging/Therapy, etc.” were performed to identify relevant citations. Moreover, we systematically discuss a few essential constraints that should be considered when designing targeted nanocarriers for PCa theranostics.

## 2. Radiometals and Chelators for Imaging and Therapy

In the design of theranostic radiopharmaceuticals, it is essential to select the appropriate isotope for pairing with the desired application [28]. Isotopes with primarily gamma (e.g., ^111^In, ^99m^Tc, ^67^Ga) or positron (e.g., ^89^Zr, ^86^Y, ^68^Ga, ^18^F, ^64^Cu, ^44^Sc) emissions are used for diagnosis via SPECT and PET imaging techniques, providing sensitive, non-invasive, and potentially quantitative images [29,30]. For applications in nanomaterial imaging, short-lived isotopes such as ^18^F are typically not suitable due to short half-life compared to the NPs with long biological half-life and clearance time. For these applications, longer-lived isotopes such as ^89^Zr, ^64^Cu, or ^111^In are preferred for imaging. Radioactive isotopes with high ionizing beta (e.g., ^212/213^Bi, ^212^Pb, ^47^Sc, ^186/188^Re, ^177^Lu, ^114m^In, ^90^Y) or alpha (e.g., ^225^Ac, ^212/213^Bi,) emissions are preferred for therapy. Targeted radioligand theranostics can deliver a highly concentrated absorbed dose for therapy to the targeted tissues. A fundamental consideration in developing radiometal-based targeted radiopharmaceuticals is the design and careful selection of an appropriate chelator that may maintain a stable coordination complex with the radiometal isotope of interest in vivo. Several of the most potent and extensively explored chelator-radiometal combinations have been summarized in Table 1 [29]. Several elements have multiple radioactive isotopes with identical chemistry that may be used for therapy or diagnostic purposes (e.g., ^86/90^Y, ^67/68^Ga, ^44/47^Sc, ^64/67^Cu), and thus can be isotopically labelled to yield similar chemical properties, biological behavior, and distribution of the radiopharmaceutical agent in vivo. While acyclic chelators such as DFO and HOPO derivatives show highly potent radiolabeling efficacy with ^89^Zr^4+^, the macrocyclic ligands DOTA and NOTA are potent chelators for multiple radionuclide isotopes of different elements due to their inherently constrained geometries and thermodynamically more favorable metal ion binding sites. In fact, DOTA is one of the current ‘‘gold standards’’ for multiple isotopes, including ^225^Ac, ^86/90^Y, ^177^Lu, ^111^In, and ^44/47^Sc [29]. Regarding radiolabeling efficiency and coordination kinetics, most acyclic chelators can undergo quick radiolabeling at room temperature with quantitative yields, whereas macrocycles may require heating. However, superior radiolabeling may not be the only parameter that should be considered while designing a new chelator or modifying existing chelators, as the structure of the radiometal–chelate complexes along with their conformations and physical properties influence the overall pharmacokinetics of radiopharmaceuticals in vivo.

## 3. PSMA and Its Targeting Ligands

**Figure 2:**
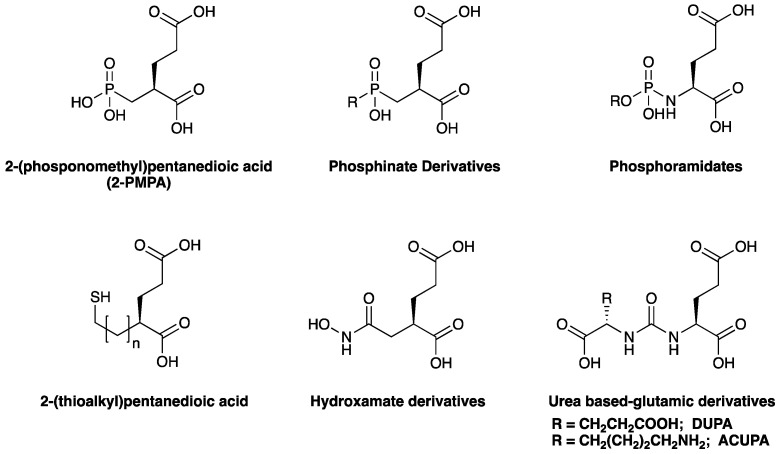
Chemical structures of main classes of small molecule-based PSMA-targeting ligands.

Reported PSMA-targeting ligands may be classified into four major groups—antibodies, aptamers, peptides, and small-molecule inhibitors. Low-molecular-weight ligands have several advantages over antibodies, including facile synthesis, tunable in vivo pharmacokinetic properties and shorter biological half-life, and biocompatibility by conjugating with a suitable linker or host. The isolation and analysis of the PSMA crystal structure was a landmark discovery providing a deep understanding of key interactions and conformations within the active site [11], which unveiled the strong affinity of the pharmacophore pocket (S1’) towards glutamate-like motifs. This facilitated the development of a large number of glutamate-based small molecule ligands that efficiently target the enzymatic pocket of PSMA with binding affinity below the nanomolar level [24]. Three major families of small molecule-based PSMA-targeting ligands include phosphonate compounds and thiols, glutamate-phosphoramidates, and glutamate-ureido derivatives (Figure 2) [24]. The ureido-glutatamate-based derivatives (DUPA and ACUPA) are currently the most widespread PSMA-targeting ligands employed in PCa imaging and therapeutic applications (Figure 2) [24]. Among them, [^68^Ga]PSMA-11 and [^18^F]DCFPyL with ureido-glutatamate-based PSMA-targeting ligands have been approved for PSMA-targeted PET imaging in men with PCa by the Food and Drug Administration (FDA) in 2020 [8]. As a landmark success, another ureido-glutatamate-based small molecule conjugate, [^177^Lu]PSMA-617 was the first therapeutic drug approved by FDA in 2022 for radioligand therapy of metastatic castration-resistant prostate cancer (mCRPC) [31]. Moreover, several other ureido-glutamate-based PSMA-targeted conjugates, including [^111^In]PSMA-I&T, [^18^F]PSMA-1007, [^225^Ac]PSMA-617, and [^177^Lu]PSMA-I&T are showing very promising response in radiological imaging and therapy of PCa and are in active clinical trials [8,24,32].

## 4. EPR Effect and Need of Targeted Nanomedicine

The effects of enhanced permeability and retention (EPR) associated with solid tumors are well known and are instrumental in the clinical efficacy of nanomedicines and macromolecular drugs [33]. The concept of EPR associated with macromolecular drugs was first proposed by Maeda et al., in 1986 [34]. In this molecular size-dependent phenomenon, macromolecules larger than 12 nm undergo passive accumulation in tumors, which may be many times higher than that in healthy tissue [35]. Solid tumors display a poorly organized vascular architecture due to excess vascular growth and permeability factors with the non-functional lymphatic drainage system [36]. Consequently, large molecules and nanomaterials may enter the tumor readily through the porous blood vessels and then accumulate as the efflux rate may be slowed by poor lymphatic drainage. Molecules that are biocompatible, stable to degradation, and remain in circulation for >6 h exhibit EPR localization in tumors [33].

**Figure 3:**
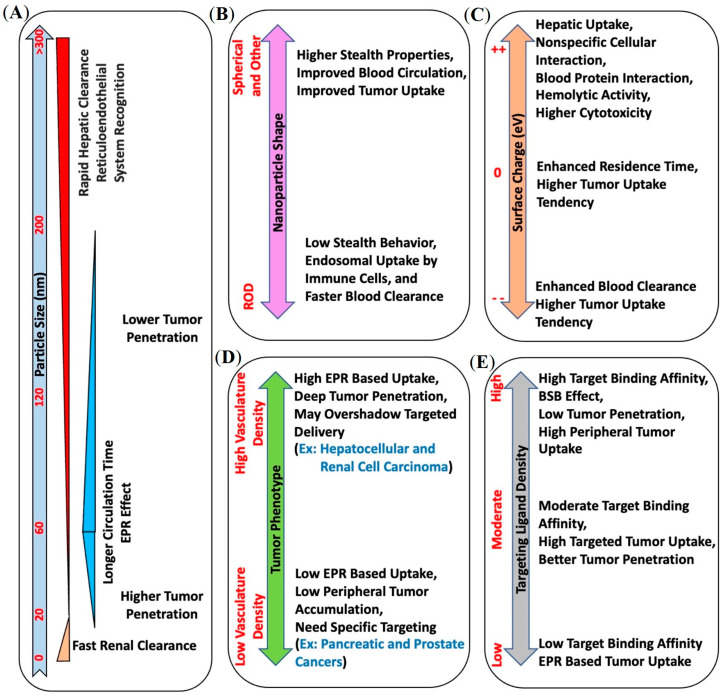
Summary of various physical and biological parameters associated with nanocarrier systems, like (**A**) size, (**B**) shape, (**C**) surface charge, (**D**) tumor phenotype, and (**E**) targeting ligand density, that potentially influence their in vivo pharmacokinetics and should be considered carefully when designing nanotheranostic systems.

The 12 nm size (40 kDa molecular weight) threshold is an important parameter as it represents the macromolecular size typically resulting in renal clearance [33,35]. Thus the plasma half-life of macromolecules larger than 12 nm increases steadily, boosting EPR-mediated tumor accumulation. The EPR effect has been well observed in nanomedicines such as polymer–drug conjugates, polymer NPs, DNA polyplexes, lipid particles, liposomes, micelles, and proteins (including IgG) [35]. SMANCS is the first EPR-based macromolecular anticancer drug by Maeda et al., approved in 1993 for clinical use [35]. However, rodent and human tumors are highly heterogeneous and possess distinct EPR effects across different tumor phenotypes [33]. For example, hepatocellular and renal cell carcinomas show relatively increased EPR-mediated tumor uptake due to high vascular density [33]. In contrast, pancreatic and prostate cancers have a low vascular density and have lower EPR-mediated tumor penetration and accumulation [33]. Overall, EPR is a complex heterogeneous phenomenon influenced by various parameters, including interstitial fluid pressure, hypoxia, vessel density, as well as nanomedicine size, shape, weight and charge (Figure 3).

While the EPR effect has proven beneficial for drug delivery, additional improvements may be realized by using molecularly targeted nanomedicines to improve tumor uptake and penetration in EPR-low phenotypes. However, interestingly, the size of nanocarriers plays a significant role in both non-targeted and targeted tumor uptake. Large-size non-targeted nanocarriers may not penetrate the bulk tumor in EPR-low phenotypes, while nanocarriers with strong target binding affinity may induce the binding site barrier (BSB) effect and block their diffusion into the bulk tumor [5,37]. These prior observations have provided a strong rationale for pursuing PSMA-targeted nanotheranostics, the topic of this review.

## 5. PSMA-Targeted Nanocarriers

Several different PSMA-targeted nanocarriers have been evaluated for radioligand imaging and therapy, including metal NPs (iron oxide), polymer self-assembly, lipid vesicles, water-soluble polymers, and dendrimers. In this review, we summarize the most relevant reports of PSMA-targeted nanocarrier systems developed thus far for radioligand imaging and therapy of prostate cancer.

### 5.1. PSMA-Targeted Metal NPs

**Figure 4:**
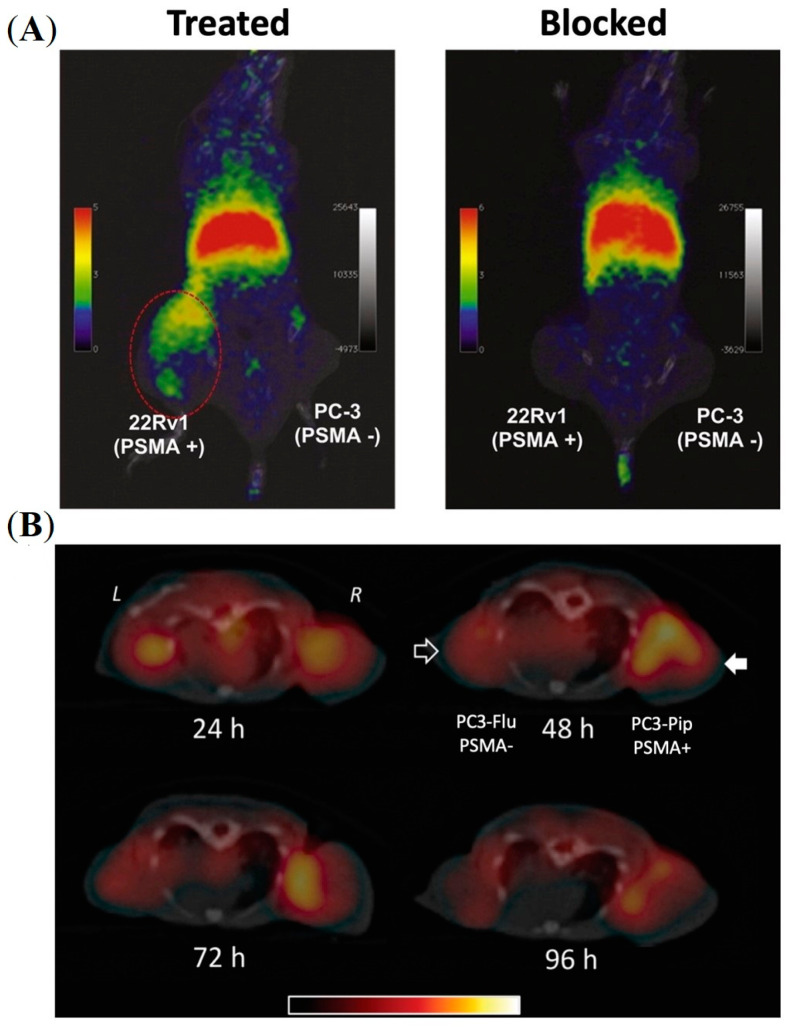
Imaging of PSMA-labelled iron oxide nanoparticles in prostate cancer murine models. (**A**) In vivo PET imaging of a mouse model bearing PSMA+ 22rv1 and PSMA—PC3 tumors at 1 h post-injection of PSMA-targeted iron oxide NPs encapsulated with DOTA- and ACUPA-conjugated PEGs. Reprinted with permission from Ref [40]. Copyright 2016, Elsevier. (**B**) In vivo SPECT-CT imaging of PSMA-targeted iron oxide NPs in mouse model bearing PSMA+ PC3-Pip and PSMA- PC3-Flu tumors over 4 days. Reprinted with permission from Ref [42]. Copyright 2015, Royal Society of Chemistry.

While PET imaging is a promising tool for both early stage as well as high-risk PCa diagnosis, multi-parametric magnetic resonance (MR) imaging has become the gold standard technique for local PCa imaging [38,39]. Magnetic iron oxide NPs are potential theranostic scaffolds for hybrid PET/MR imaging. However, one of the key challenges in clinical translating is the requirement of sufficiently high amount of paramagnetic metal to allow detection with MRI devices. By using a straightforward one-pot synthesis technique, Moon and co-workers developed PSMA-targeted iron oxide (Fe_3_O_4_) NPs for hybrid PET/MR imaging [40]. Iron oxide NPs of average size ~11 nm were formulated by encapsulating with DOTA- and ACUPA-conjugated PEG derivatives using ultrasonication, and were efficiently radiolabeled with ^68^Ga for PSMA-targeted PET/MR imaging. In vivo PET/MR imaging was performed in the BALB/c mouse model with PSMA+ 22rv1 and PSMA-PC-3 dual xenografts, showing improved MR resolution with PSMA-targeted accumulation of the NPs in 22rv1 tumors at 1 h post-injection (Figure 4A). Although the spatial resolution of PET obtained was lower than that of anatomical MR images, PET imaging could successfully provide quantitative information on drug delivery. Representing complementary tools, the formulation of a dual-modality nanosystem integrated with PET and MR imaging agents represents a promising advance in PCa diagnosis.

Another iron oxide-based NPs system was developed by Liolios et al. for its potential application in PCa diagnosis using PET/MR imaging [41]. By using a sol-gel technique, the co-precipitated form of ferrous and anhydrous ferric oxide was surface functionalized with (3-aminopropyl)triethoxysilane (APTES) or 3-mercaptopropyltrimethoxysilane (MPTES). The resulting NPs, covalently conjugated with pharmacophores targeting PSMA (ACUPA) and gastrin-releasing peptide receptors (GRPR; bombesin peptide) via -SH or –NH_2_ reactive functional groups, had an average size of around 73.6 nm or 66.5 nm, respectively. Following a direct ^68^Ga-labelling process, targeted and non-targeted NPs with either thiol or amine linkers were developed. The surface-modified iron oxide NPs were evaluated in cells expressing PSMA (LNCaP) and GRPR (PC-3) showing high target binding affinity (LNCaP: Kd = 11.49 nM, PC-3: Kd = 28.27 nM) with low toxicity, and thus were identified as a potential candidate for multimodal PET/MR imaging.

Azad and co-workers developed PSMA-targeted SPECT and optical imaging agents based on a iron oxide NPs and evaluated their PSMA-targeting efficacies in a mouse model bearing PSMA+ PC3-Pip and PSMA- PC3-Flu dual xenografts (Figure 4B) [42]. The free amine groups were functionalized with either the optical agent (IRDye 800CW) or a radiometal chelator (DTPA) and were conjugated along with PSMA-targeted ACUPA ligands. The NPs were coated with PEG_1000_ to improve immune evasion and blood circulation time with increasing nanoparticle size. SPECT imaging and ex vivo biodistribution demonstrated high PSMA-targeted tumor accumulation in PC3-Pip xenografts (4.29%ID/g) at 48 h post-injection. Similarly, optical images also demonstrated target-specific accumulation at 4 h post-injection. Interestingly, at longer timepoints up to 96 h, the accumulation of NPs was significantly reduced in PC3-Pip tumors to 1.99%ID/g, whereas the nonspecific tumor accumulation in PC3-Flu tumors increased to 1.63%ID/g, which suggests EPR-mediated tumor accumulation of the NPs at later timepoints.

In addition, several other PSMA-targeted metal nanoparticle systems, such as ^177^Lu-labeled lutetium oxide (Lu_2_O_3_) NPs [43,44] and ^223^Ra-labeled NaA zeolite NPs [45,46], have been developed and evaluated to treat prostate cancer. These reports also demonstrated chelator-free radiolabeling of theranostic isotopes into the respective metal NP cores. The synthesis of lutetium oxide NPs and their radiolabeling was carried out simultaneously by adding the appropriate fraction of radioisotope ^177^Lu at the time of NP formulation, whereas the radiolabeling of ^223^Ra to the NaA nanozeolite was carried out by exchanging Na^+^ for ^223^Ra^2+^ cation. Although these chelator free nanozeolites demonstrated promising PSMA-targeted cell uptake, they failed to show any PSMA-targeted tumor uptake with high accumulation in the lungs, liver, spleen, and bones, which could be due to the large particle size [46]. However, the lutetium oxide NPs demonstrated selective toxicity to malignant tumors without any histological changes in healthy tissues, thus supporting their potential for clinical translation [44].

### 5.2. PSMA-Targeted Amphiphilic Block Copolymers

Amphiphilic block copolymers are potent drug delivery platforms that readily form NPs suitable to accommodate high payload quantities with controlled release. The block copolymer of polylactic glycolic acid (PLGA)/polylactic acid (PLA) and polyethylene glycol (PEG) represents an ideal combination with a hydrophobic core covered with an outer hydrophilic corona, protecting the NPs from immune surveillance [47,48]. These polymers are also safe for human use. The ability to control nanoparticle size is crucial to overcoming physiological barriers in vivo, which may be achieved by optimizing formulation parameters.

In pioneering studies, Farokhzad and co-workers evaluated the role of PSMA-targeting amphiphilic block copolymers. They performed a thorough evaluation of PLGA-b-PEG polymer NPs and demonstrated the effects of formulation parameters on nanoparticle size [49]. It was observed that the nanoparticle size decreased with an increase in the water fraction, whereas the size increased with an increase in polymer and payload concentration. Furthermore, NPs conjugated with PSMA-targeted A10 RNA aptamer and loaded with the tracing agent ^14^C-paclitaxel were evaluated for their targeting efficacy in PSMA+ LNCaP xenografts. The targeted NPs demonstrated a 3-fold higher accumulation at 24 h post-injection compared to their non-targeted versions. Despite sub 1% ID tumor uptake, the authors attributed the 3-fold higher accumulation of the A10 RNA aptamer-conjugated NPs to target-specific tumor uptake. The NPs were speculated to suffer from the burst effect, releasing a large quantity of the ^14^C-paclitaxel payload. Decreased tumor uptake at 6 h and 24 h time points was possibly due to diffusion of the payload at later time points.

**Figure 5:**
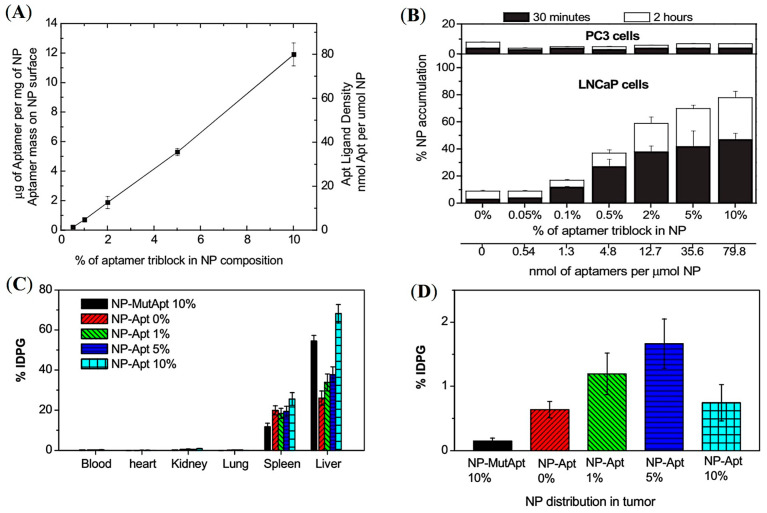
Development of PSMA-targeted amphiphilic block copolymers using aptamers as a targeting ligand. (**A**) Quantification of aptamer ligand density on the PLGA-b-PEG nanoparticle surface. (**B**) Cell uptake assay of ^3^H-labelled NPs in LNCaP and PC3 cells. (**C**,**D**) Ex vivo biodistribution of NPs with different % of aptamer conjugated PLGA-b-PEG polymer in LNCaP tumor-bearing mice administered by retro-orbital injection. Reprinted with permission from Ref [50]. Copyright 2008, National Academy of Science.

One central challenge is designing targeted polymer NPs with controlled drug release. Another report by Farokhzad and co-workers demonstrated that the molecular mass of the PEG is the key factor in influencing the nanoparticle size, whereas increasing the molecular size of PLGA prolonged the drug release rate [50]. A series of PSMA-targeted NPs were developed using the optimum length of PLGA-b-PEG chains conjugated with PSMA-targeting aptamer (A10). The aptamer densities were varied by mixing different ratios of PLGA-b-PEG-A10 and PLGA-b-PEG (Figure 5A). PLGA-b-PEG-A10 with a trace amount of ^3^H-labelled PLGA was incorporated to formulate tritium-labelled NPs and enable biodistribution studies. A gradual increase in the accumulation of NPs was observed by increasing the fraction of PLGA-b-PEG-A10 up to 10% in PSMA+ LNCaP cells (Figure 5B). Conversely, ex vivo organ biodistribution of the targeted NPs in LNCaP tumors showed maximum tumor uptake at 5% aptamer density (Figure 5C,D). In contrast, with a further increase to 10% aptamer density, a decrease in tumor accumulation was observed in the tumors, which was similar to the non-targeted NPs. This demonstrated the EPR-mediated passive accumulation of the NPs without any influence of the PSMA-targeted aptamers (Figure 5D). Interestingly, the increased aptamer surface density reduced immune system evasion and caused increased hepatic clearance of the NPs. This study highlights the significance of precisely tuning the ratio of targeting ligand to the nanoparticle to maximize in vivo uptake of the polymer.

**Figure 6:**
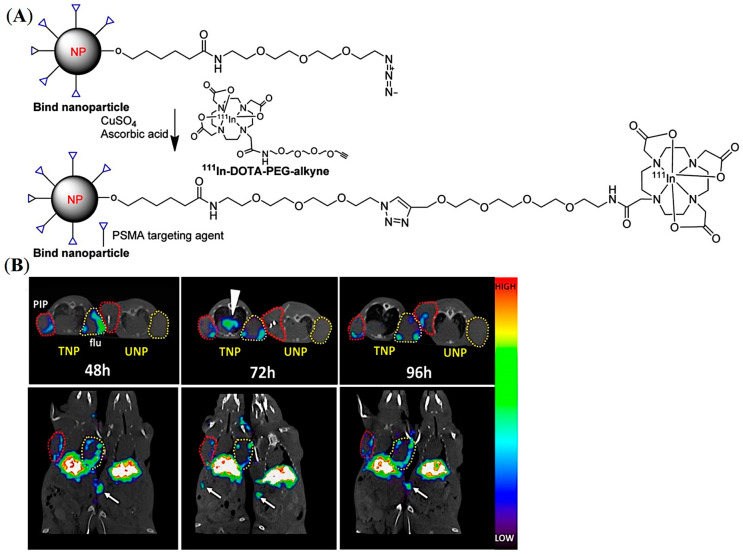
Development of PSMA-targeted PLA-PEG NPs for imaging of prostate cancer in mouse models. (**A**) Representative structure of the PSMA-targeted PLA-PEG NPs. (**B**) In vivo SPECT-CT of ^111^In-labelled PSMA-targeted and non-targeted NPs in PSMA expressing PC3-Pip (red circles) and PSMA negative PC3-Flu (yellow circles) tumor-bearing mice model up to 96 h. White arrows show prominent spleen uptake. Reprinted with permission from Ref [51]. Copyright 2017, American Chemical Society.

A similar pattern of tumor accumulation of PLA-b-PEG NPs was demonstrated by Pomper and co-workers beyond 24 h post-injection by using small molecule-based PSMA-targeting ligands (ACUPA) in a mice model bearing dual xenografts of PSMA+ PC3-Pip and PSMA- PC3-Flu tumors (Figure 6A) [51]. The NPs were conjugated with the ^111^In radiometal chelator DOTA and near-infrared (NIR) dye IRDye680RD for simultaneous SPECT and fluorescence imaging. The SPECT/CT imaging, fluorescence imaging, and organ biodistribution demonstrated almost no influence on the PSMA-targeting ligands at 48 h post-injection, while very modest increments of the targeted NPs in PC3-Pip tumors were observed at 96 h post-injection (Figure 6B). The authors hypothesized that the observed tumor accumulation may be due to tumor-associated macrophages and the EPR effect. It was also claimed that, although there is a single genetic difference between PC-3-Pip and PC3-Flu cells, the subcutaneous PC3-Pip tumors possess a significantly higher quantity of CD68-expressing macrophages and CD31+ vasculature than that of PC3-Flu tumors, which may be responsible for the modest increase in uptake of the targeted NPs in PC3-Pip xenografts.

Preliminary studies developing the prostate cancer-targeted PLGA-b-PEG NPs to deliver optimum boron content for boron neutron capture therapy (BNCT) have been conducted. BNCT is a localized cancer treatment technique using selective accumulation of boron-10 atoms in tumor tissue followed by low-energy neutron beam exposure to induce a nuclear fission reaction and generate a-particles [52]. These nuclear fragments possess sufficient energy within 6–9 mm to destroy cancer cells [53]. Localized treatment using BNCT potentially reduces systemic toxicity and side effects. However, the primary challenge associated with this technique is the lack of efficient boron delivery agents. BNCT requires a high boron content (>20 μg/g tissue) with more than 5-fold higher target-to-background to effectively kill cancer cells [54]. BNCT has been used in the treatment of neck and head cancers and glioma, but despite preliminary investigations, this technology has never been successfully applied in PCa, due to the lack of suitable targeting probes [55].

**Figure 7:**
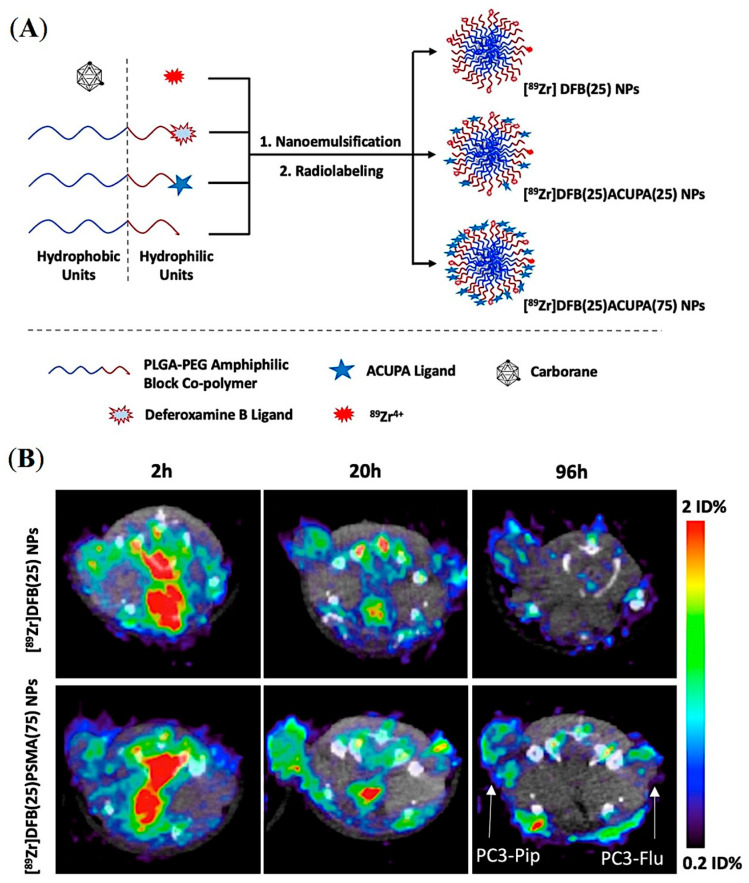
Design and preliminary evaluation of carborane-loaded, PSMA-targeted PLGA–PEG NPs for imaging and treatment of prostate cancer using BNCT. (**A**) Graphical presentation of carborane-loaded PLGA–PEG NPs radiolabeled with ^89^Zr for targeted boron delivery. (**B**) Axial μPET/CT imaging of mice bearing dual xenografts of PC3-Pip and PC3-Flu at different time points. Reprinted with permission from Ref [57]. Copyright 2021, American Chemical Society.

A series of carborane-labelled ACUPA ligands were designed and synthesized, demonstrating excellent PSMA binding affinity in vitro [56]. Unfortunately, these small molecular probes were only able to deliver up to 4.2 μg boron/gram tumor in vivo, which was around 5-fold less than the required boron content for efficient BNCT. Inspired by other reports that demonstrate more than 50 μg boron/gram tumor using carborane-loaded PLGA NPs by simply relying on the EPR effect, it was hypothesized that coupling a PSMA ligand to carborane-loaded NPs would increase boron delivery to PCa [57]. As presented in Figure 7A, DFB- and ACUPA-conjugated PLGA-b-PEG amphiphilic block copolymers were prepared and were nano-emulsified to form NPs loaded with carborane spontaneously. Three different NPs were formulated without or with different % weight of ACUPA-conjugated PLGA–PEG. The DFB conjugated NPs were radiolabeled with ^89^Zr and showed good PSMA-targeted cell binding affinity in PSMA+ PC3-Pip cells. However, no targeted boron delivery was observed in those NPs due to the fast release of carborane from the nanoparticle core.

In vivo PET imaging and organ biodistribution of the ^89^Zr-labelled NPs was performed in mice models containing PSMA+ PC3-Pip and PSMA- PC3-Flu dual xenografts (Figure 7B). Unexpectedly, although the PC3-Pip/blood ratio was around 25, these NPs did not show any PSMA-targeted delivery with tumor uptake of ~1%ID. Despite the presence or absence of the PSMA-targeting ACUPA ligands, a 2-fold higher tumor uptake in PC3-Pip than in the PC3-Flu was observed, which may be due to EPR-mediated passive uptake [58]. On the other hand, those NPs showed a fast release of carborane from the PLGA core, which resulted in inefficient boron delivery to tumors. Although this kind of PLGA–PEG NPs have been demonstrated to have excellent loading and delivery of drug-like docetaxel [47,50,59], they may not effectively deliver hydrophobic molecules such as carborane. Several improvements, such as crosslinked amphiphilic polymer NPs and alkyl chain-conjugated carboranes, may be employed to enhance boron loading and release kinetics to achieve optimum boron delivery. Overall, the formulation of an organic polymer-based targeted nanoparticle platform requires a delicate balance between individual physicochemical properties and needs to be experimentally determined as well as precisely and reproducibly engineered for in vivo success.

### 5.3. PSMA-Targeted Liposomes

**Figure 8:**
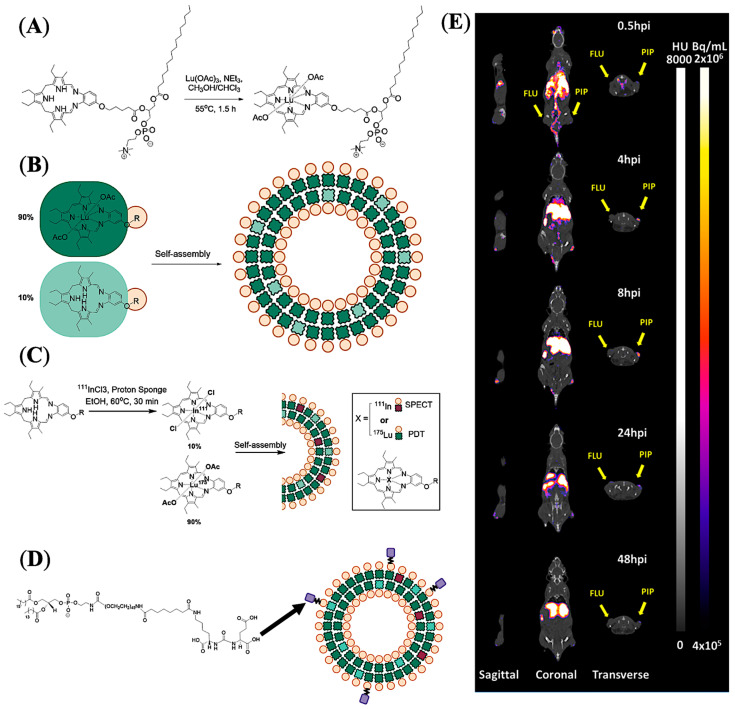
Imaging and treatment of prostate cancer in mouse models using PSMA-targeted liposome-like texaphyrin NPs. (**A**) Synthetic scheme showing chelation of lutetium with texaphyrin−lipid. (**B**) Formulation and representation of nanotexaphyrin self-assembly. (**C**) Radiolabeling conditions and schematic representation of ^111^In/Lu-labelled nanotexaphyrin. (**D**) Schematic representation of PSMA-targeting ligand (ACUPA) conjugated ^111^In/Lu–nanotexaphyrin. (**E**) mSPECT/CT images of a mouse model bearing dual xenografts of PC3-Pip and PC3-Flu. Reprinted with permission from Ref [61]. Copyright 2022, American Chemical Society.

Liposomes represent a unique class of nanostructures composed of (phospho)lipids and cholesterol enabling encapsulation of both hydrophilic and lipophilic drug molecules [60]. Amphiphilic lipid molecules readily self-organize to form nanovesicles in aqueous media, in which the lipid bilayer membrane can entrap hydrophobic molecules, and the internal aqueous region can hold large quantities of hydrophilic molecules including macromolecules for efficient drug delivery. Zheng and co-workers developed PSMA-targeted liposome-like NPs through nanotexaphyrin−lipid self-assembly, in which nanotexaphyrin may allow the chelation of both ^111^In and Lu for simultaneous SPECT/CT imaging and photodynamic therapy (PDT) [61]. The nanotexaphyrin was formulated using ethanol injection of a dried lipid film into an aqueous PBS buffer followed by extrusion after 30 min stirring at 60 °C. The dried film was prepared with a composition of 27.5% mol dipalmitoylphosphatidylcholine (DPPC), 27.5% mol 9:1 ratio Lu-texaphyrin-lipid:texaphyrin-lipid, 40% mol cholesterol, and 5% mol PEGylated phospholipid (DSPE-PEG_2000_) (Figure 8A,B). Interestingly, the authors successfully developed a rapid and robust microfluidic system that efficiently facilitated the chelation of ^111^In/^175^Lu to nanotexpahyrin with high yield without affecting texaphyrin−lipid self-assembly (Figure 8C). The optimized metalated nanotexaphyrin of around 100 nm displayed excellent photo and chemical stability with a favorable blood circulation half-life (t_1/2_ = 6.6 h), and demonstrated potent singlet oxygen generation. Urea-based PSMA-targeting lipid NPs were prepared by adding 5% mol ACUPA-lipid conjugates, and tumor accumulation of the NPs was evaluated by both SPECT/CT imaging and NIR fluorescence imaging and ex vivo organ biodistribution in a mice model bearing PSMA+ PC3-Pip and PSMA- PC3-Flu xenografts (Figure 6D,E). The texaphyrin-phospholipid NPs conjugated with PSMA-targeting ACUPA ligands enabled specific accumulation in PSMA+ PC3-Pip tumors and reached the peak (4.5%ID/g) at the 8 h time point with 2-fold higher uptake than PSMA- PC3-Flu tumors. However, at the 48 h timepoint, the accumulation in PC3-Pip decreased to 3%ID/g, which was very close to the 2.2%ID/g uptake of NPs in PC3-Flu tumors, which was also comparable to the 2.5%ID/g in KB (HeLa derivative) subcutaneous tumors [62]. These results suggest that the tumor accumulation at earlier time points was driven by PSMA receptors, whereas the enhanced permeability and retention (EPR) effect became more dominant at later time points. In addition, with laser irradiation at 8 h post-injection, these PSMA-targeting NPs showed a potent PDT effect and successfully inhibited the growth of PC3-Pip tumors. Overall, this study demonstrates potent theranostic capabilities of the metal chelation-driven texaphyrin NPs, which, in combination with PSMA-targeting ligands, may enable PCa imaging and therapy.

**Figure 9:**
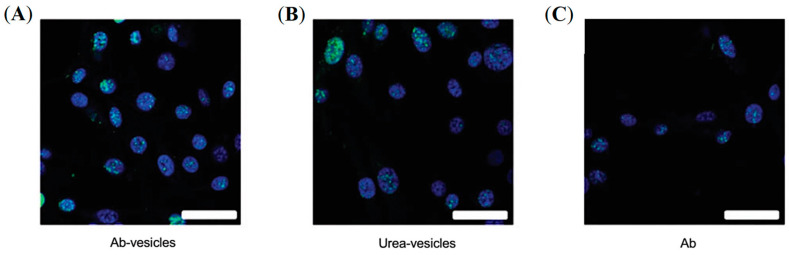
Treatment of prostate cancer using PSMA-targeted, ^225^Ac-labelled liposomes. Immunofluorescent images of g-H2AX foci (green) in cell nuclei (blue) upon treatment with Ab-targeted vesicles (**A**), urea-targeted vesicles (**B**), and radiolabeled Abs (**C**). Scale bar, 40 mm. Reprinted with permission from Ref [63]. Copyright 2016, American Association of Cancer Research.

A similar approach was employed to develop PSMA-targeted lipid vesicles radiolabeled with a-emitting ^225^Ac for PCa therapy [63]. Two different targeted vesicles were prepared by either conjugating PSMA-targeting lysine–glutamate urea-based ligands or antibodies, and their cell killing efficacy was compared with a PSMA-targeting antibody (Figure 9). For radiolabeling ACUPA-conjugated liposomes, the vesicles were allowed to encapsulate citrate buffer containing DOTA, followed by radiolabeling with ^225^Ac at 80 °C. After 1 h of incubation, the free ^225^Ac was trapped by adding DTPA and purified by size exclusion chromatography. For the antibody-conjugated liposomes, DOTA-Isothiocyanate was first chelated with ^225^Ac followed by its conjugation to the antibody. Fluorescence confocal microscopy images showed that the PSMA-targeted vesicles localize close to the cell nucleus, unlike the preferential localization of targeted antibodies around the plasma membrane, which resulted in increased levels of dsDNAs with around 3-fold higher cell killing efficacy of the PSMA-targeted vesicles. The lipid vesicles were of 107 ± 5 nm size with either 31 ± 9 antibodies or 368 urea-based PSMA-targeting ligands and thus may provide further opportunity to improve cell internalization and therapeutic efficacy by optimizing the ligand density and size of NPs.

**Figure 10:**
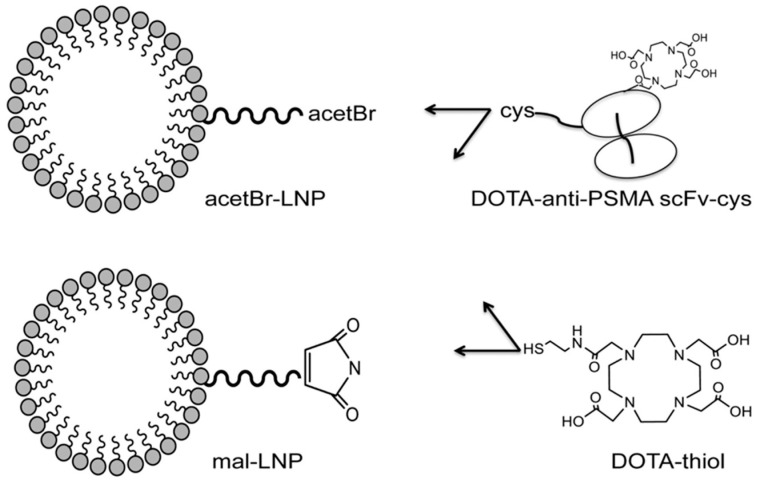
Representative constructs for scFv-conjugated lipid vesicles. Reprinted with permission from Ref [64]. Copyright 2017, Elsevier.

Recombinant single-chain antibodies (scFvs) are also highly potent targeting ligands, but undergo rapid clearance due to their relatively small molecular weights (below 30 kDa). Wong and co-workers demonstrated the conjugation of PSMA-targeting scFv to the DSPE-PEG_2000_-based lipid vesicles of 12 nm average size, resulting in superior tumor accumulation compared to that of scFv alone and non-targeted lipid vesicles [64]. As shown in Figure 10, by using different linkers, two targeted lipid vesicles were prepared and compared with their respective non-targeted lipid vesicles and the targeting ligand alone. All the constructs were radiolabeled with ^64^Cu, and PET imaging and organ biodistribution were carried out in a NOD/SCID mice model bearing PSMA+ LNCap tumors. It was observed that the conjugation of lipid vesicles of around 12 nm not only improved the blood circulation time of scFvs, but also significantly enhanced the targeted tumor accumulation of lipid vesicles up to 1.6–2 fold compared to the non-targeted vesicles and the targeting ligand alone. In another report, the authors explored the targeted delivery of the lipid vesicles covalently conjugated with chemotherapeutic drugs (doxorubicin or auristatin) and a bivalent anti-PSMA diabody [65]. The lipid vesicles conjugated with PSMA-targeted diabodies demonstrated as high as 15%ID/g uptake in PSMA+ LNCap xenografts with reasonable therapeutic response with auristatin-conjugated lipid vesicles.

### 5.4. PSMA-Targeted Nanoplex

**Figure 11:**
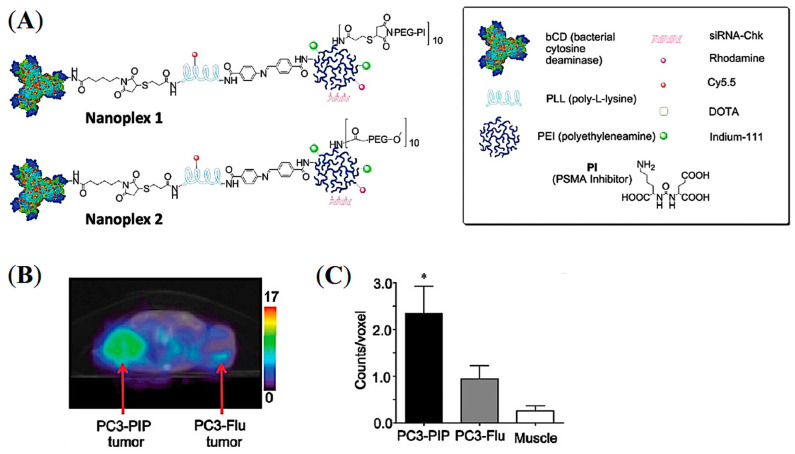
PSMA-targeted nanoplex for combined imaging and treatment of PCa using a prodrug enzyme strategy and siRNA. (**A**) Schematic representation of the PSMA-targeted nanoplex 1 and 2. (**B**) Transaxial mSPECT/CT images of the targeted ^111^In-labelled nanoplex 1 at 48 h post-injection in SCID mouse bearing PC3-PIP and PC3-Flu tumors. (**C**) ROI on tumors and muscle at 48 h post-injection (*n* = 4, * *p* < 0.05). Reprinted with permission from Ref [67]. Copyright 2012, American Chemical Society.

Small interfering RNA (siRNA)-mediated suppression of specific target mRNAs has substantial potential in cancer treatment by down-regulating cancer-specific pathways [66]. Combining the prodrug enzyme therapy strategy with siRNA use may enhance cancer-selective therapy without systemic toxicity. As a proof of concept, Bhujwalla and co-workers demonstrated a PSMA-targeted nanoplex platform for PCa theranostics by delivering a prodrug enzyme along with siRNA [67]. As shown in Figure 11A, the nanoplexes were constructed with covalently linked major components including a near-infrared (NIR) fluorescent probe Cy5.5, a prodrug-activating enzyme bacterial cytosine deaminase (bCD), and radiometal ^111^In chelator DOTA for SPECT imaging. The prodrug enzyme bCD was employed to convert the nontoxic prodrug 5-fluorocytosine (5-FC) to 5-fluorouracil (5-FU). The siRNA was associated with the polyethyleneimine (PEI) dendrimer through electrostatic interactions to down-regulate choline kinase (Chk) that can enhance the effect of 5-FU. Moreover, PSMA-targeting ACUPA ligands were also tethered to PEI for PCa-targeted imaging and drug delivery. The conjugation of multiple imaging modalities is highly advantageous to evaluate the in vivo and microscopic distribution of drugs in cells, cellular organelles, and ex vivo tissue samples.

The PSMA-targeted Nanoplex 1 and non-targeted Nanoplex 2 were tested in PSMA+ PC3-Pip and PSMA- PC3-Flu cells and tumor xenografts, in which the Nanoplex 1 demonstrated enhanced uptake in PC3-Pip cells and tumors. SPECT/CT images of ^111^In-labelled Nanoplex 1 showed increased accumulation in PC3-Pip tumors at 48 h post-injection (Figure 11B,C). This finding suggests that efficient PSMA-targeted tumor accumulation with suppressed EPR-mediated passive accumulation was achieved. In addition, the major advantage of having non-invasive imaging reporters on the nanoplex is to visualize the nanoplex distribution effectively and to choose the precise timepoint(s) for prodrug enzyme injection. The developed targeted nanoplex with multimodality imaging reporters together with prodrug enzyme and siRNA may be advantageous in the theranostic application in metastatic PCa and may also be extended to other cancer subtypes and therapeutic targets. Although there was high uptake of the nanoplex in the liver and kidneys, down-regulation of the silenced gene Chk showed negligible toxicity on nonmalignant cells.

### 5.5. PSMA-Targeted Multivalent Dendrimers and starPEG Nanocarriers

**Figure 12:**
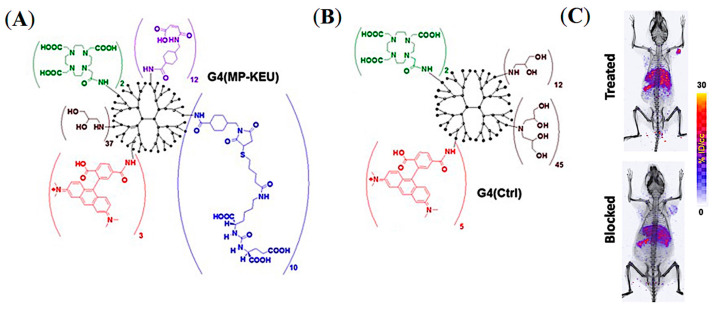
Representative chemical structures of (**A**) PSMA-targeted G4(MP-KEU) and (**B**) control G4(Ctrl) dendrimers. (**C**) Volume-rendered mPET/CT images of NOD-SCID mice model bearing dual xenografts of PC3-Pip and PC3-flu. Reprinted with permission from Ref [68]. Copyright 2019 American Chemical Society.

**Figure 13:**
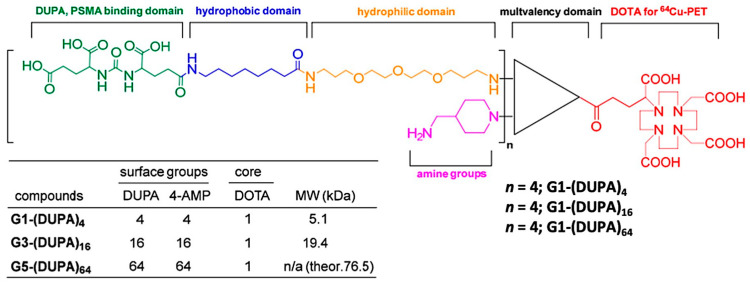
Representative chemical structures of triazine dendrimers conjugated with PSMA-targeting DUPA ligands and radiometal chelator DOTA. Reprinted with permission from Ref [5] Copyright 2019, MDPI.

As discussed previously, the pharmacokinetics of both active and passive tumor targeting is strongly influenced by nanocarrier size, hydrophobicity, and net surface charge (Figure 3). Compared to polymer-based NPs and antibody−drug conjugates (ADC), dendrimers and water-soluble polymers are advantageous because they provide a more facile platform to control their physicochemical properties, including the number of reactive terminal groups for drug conjugation and overall size. Pomper and co-workers demonstrated the PSMA-targeted tumor uptake of a 27.3 kDa generation 4 (G4) polyamidoamine dendrimer, G4(MP-KEU), conjugated with ACUPA ligands, that showed very high PSMA-targeted tumor accumulation compared to the control dendrimer, G4(Ctrl), of 23.2 kDa without any ACUPA ligands (Figure 12) [68]. It was observed that the targeted dendrimer, [^64^Cu]G4(MP-KEU), with a higher molecular weight and size, showed 6-fold higher blood half-life compared to non-targeted [^64^Cu]G4(Ctrl). Relatively high liver and spleen uptake of [^64^Cu]G4(MP-KEU) as compared to [^64^Cu]G4(Ctrl) was confirmed to be due to transchelation of ^64^Cu to endogenous ceruloplasmin proteins. [^64^Cu]G4(Ctrl) showed significantly higher uptake in PSMA- PC3-Flu tumors as a result of passive uptake (Figure 12C). This finding may also be attributed to the fact that the PSMA-targeting moieties in the G4(MP-KEU) dendrimer are responsible for the extended blood circulation because of increased molecular size and weight. Thus, an elevated background uptake of the targeted dendrimer were observed, including in the PC3-Flu tumor, as a result of EPR-mediated passive uptake (Figure 4a and Figure 13).

In contrast to G4 polyamidoamine dendrimers, Simanek and co-workers demonstrated a comparative in vivo analysis of PSMA-targeted G1, G3, and G5 triazine dendrimers that provides more clarity on how the size of the nanocarriers strongly influences the in vivo pharmacokinetics of both active and passive tumor uptake [5]. The triazine dendrimers were tethered to 4, 16, or 64 copies of PSMA-targeting 2-[3-(1,3-dicarboxypropyl)-ureido]pentanedioic acid (DUPA) ligands and all the dendrimer conjugates were radiolabeled with ^64^Cu for in vitro and in vivo analysis (Figure 13). While 5.1 kDa dendrimers with 4 DUPA ligands showed very high PSMA-targeted uptake in PC3-Pip cells, G5 dendrimers with 64 DUPA ligands showed high nonspecific binding to PC3-Flu cells. Despite the increase in the number of DUPA ligands from 4 to 64, the uptake in PC3-Pip and PC3-Flu tumors became similar due to an increase in the dendrimer size from 5.1 kDa to 76.5 kDa. These results demonstrated the overwhelming EPR effect associated with large-size nanocarriers, which is efficient enough to nullify the PSMA-targeted tumor uptake despite the multivalency of larger dendrimers (Figure 3A). However, compared to the high PSMA-targeted uptake of previously discussed 27.3 kDa polyamidoamine dendrimers [68], the lower uptake of these small-size triazine dendrimers was observed. Overall, these results demonstrated that nanocarriers of optimum size and suitable PSMA-targeting ligands may be a potential scaffold to improve PSMA-targeted uptake in EPR-low PCa for imaging and therapy.

**Figure 14:**
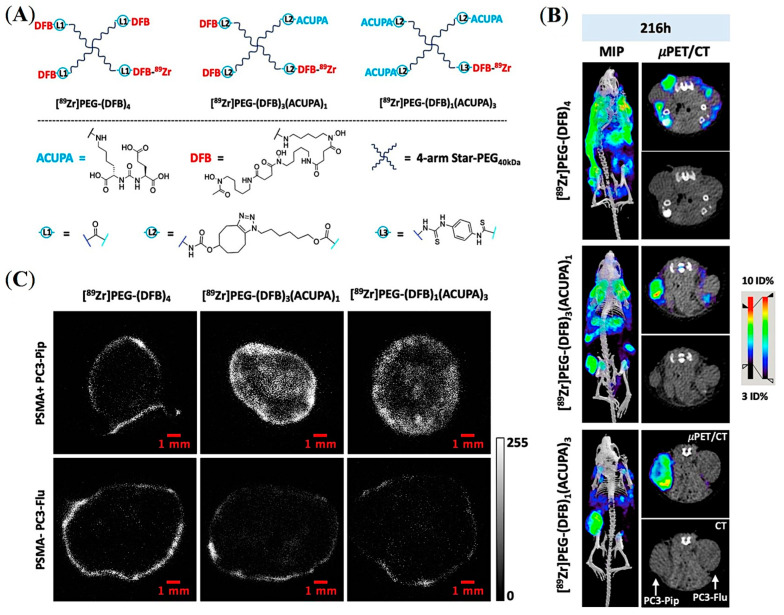
Development of PSMA-targeted starPEG nanocarriers for prostate cancer imaging. (**A**) Representative chemical structures of ^89^Zr-labelled PEG nanocarriers without and with different numbers of PSMA-targeting ACUPA ligands. (**B**) Maximum intensity projection (MIP) μPET/CT, axial μPET/CT, and axial CT images obtained at 216 h following administration of ^89^Zr-labelled nanocarriers in mice model bearing PC3-Pip and PC3-Flu dual xenografts. (**C**) Autoradiography images of tumor slices were collected after 216 h post-injection of the ^89^Zr-labelled nanocarriers. Reprinted with permission from Ref [69]. Copyright 2022, American Chemical Society.

Considering the threshold size for renal clearance (Figure 3A), Beckford-Vera and colleagues evaluated a series of non-targeted 4-armed starPEG nanocarriers of 40 kDa. The ^89^Zr-labelled PET tracer demonstrated EPR-mediated high tumor uptake and retention in HT-29 and MX-1 tumor models [58], whereas they showed low uptake in PC3-Pip PCa xenograft [58,69]. Notably, various other macromolecules and NPs have shown low EPR-mediated tumor uptake in preclinical human PCa models such as PC3, DU-145, and CWR22rv1 [5,6,7]. Meher et al. hypothesized and demonstrated that conjugation of ACUPA ligands to those starPEG nanocarriers can improve tumor uptake of EPR-low PCa models with enhanced tissue penetration and retention [69]. Three 4-arm StarPEG nanocarriers with or without distinctive numbers of PSMA-targeting ACUPA ligands were designed and synthesized (Figure 14A). The Deferoxamine B (DFB) radiometal chelator was conjugated to the nanocarrier for ^89^Zr labelling, and the radiolabeled conjugates were evaluated in PC3-Pip and PC3-Flu cell lines and xenografts, respectively. The ^89^Zr-labelled star-PEG with an increasing number of ACUPA ligands per molecule showed significantly higher in vitro PSMA binding affinity in PC3-Pip cells. However, the in vivo PET images and organ biodistribution demonstrated the highest PC3-Pip tumor uptake of targeted nanocarriers with one ACUPA ligand (9.64 ± 0.87 for [^89^Zr]PEG-DFB_3_-ACUPA_1_ and 6.69 ± 1.24 for [^89^Zr]PEG-DFB_1_-ACUPA_3_) (Figure 14B). The lower tumor uptake of the nanocarrier with three ACUPA ligands compared to the nanocarrier with one ACUPA ligand may be due to the BSB effect observed in large-size targeted nanodrugs with strong binding affinity (Figure 3A). In contrast, the non-targeted nanodrug [^89^Zr]PEG-DFB4 showed 5.75 ± 0.74 ID% of EPR-mediated tumor uptake. The autoradiography images showed highly prominent deep-tumor penetration of targeted nanocarriers in PSMA+ PC3-Pip xenograft (Figure 14C). In contrast, non-penetrating low tumor uptake was witnessed for the non-targeted nanocarriers in both PC3-Flu and PC3-Pip xenografts. Overall, an enhancement in the PC3-Pip tumor uptake in the presence of ACUPA ligands in the administered nanocarriers was observed with improved retention time, tissue penetration, and PC3-Pip/blood ratio, which may be employed in therapeutic applications.

## 6. Perspectives and Conclusions

Nanocarriers possess several advantages in biomedical applications, compared to conventional drugs, such as longer biological half-life and bioavailability, higher surface-to-volume ratio, and multiple reactive terminals, allowing versatile encapsulation and surface functionalization of suitable theranostic payloads. However, it has been challenging to design optimum nanocarriers for their biomedical applications as various physical parameters such as shape, size, and surface charge drastically influence the interaction of nanostructures at the cellular and molecular level. The overall influence of various physical parameters and their in vivo pharmacokinetics are summarized in Figure 3. For example, rod-shaped cationic nanostructures are cleared through endosomal uptake by immune system cells, possibly due to their similarity to rod-shaped bacteria [70,71]. Likewise, NPs with positively charged surfaces realize higher uptake in liver hepatocytes and become more cytotoxic than negatively charged and neutral NPs [72,73]. In contrast, negatively charged nanostructures show a higher preference for tumor accumulation with lower toxicity [73]. Self-assembled supramolecular NPs are prone to aggregation and protein opsonization and may attract immune response with rapid clearance from the bloodstream, limiting their bioavailability.

The size of the nanocarriers, whether targeted or non-targeted, has a profound influence on their in vivo tumor accumulation and retention. As previously discussed, to improve bioavailability and target-specific tumor uptake, the size of the nanocarriers should be larger than 5 nm [74,75]. However, with the nanocarrier size above 12 nm, EPR-mediated passive accumulation becomes more prominent and PSMA-targeted uptake is suppressed (Figure 3A) [4,58]. Depending on the tumor phenotype, it may result in low, peripheral accumulation (Figure 14C). Unfortunately, most of the PCa xenografts possess less permeable vasculature resulting in EPR-mediated low tumor accumulation, which is inadequate for optimum therapeutic response [6,7,69]. As observed by Simanek and co-worker, increasing PSMA-targeting ligands from 4 to 64 copies in a dendrimer-based macromolecular system resulted in similar low non-penetrating accumulation in both PSMA- PC3-Flu and PSMA+ PC3-Pip tumors due to an increase in the dendrimer size from 5 kDa to 76 kDa [5]. In contrast, the G4 dendrimer of around 27.3 kDa molecular weight conjugated with only 10 copies of PSMA-targeting ligand demonstrated very high specific tumor accumulation in PC3-Pip compared to PC3-Flu [68]. These reports suggest that conjugating a higher number of targeting ligands may not always increase target-specific tumor retention. Apart from EPR-mediated passive accumulation, the BSB effect often influences the target-specific accumulation of large-size nanocarriers and leads to non-penetrating tumor uptake due to very high target binding affinity to the cells adjacent to blood vessels blocking further tumor penetration (Figure 3C) [37]. In other words, both passive and active tumor uptake of nanocarriers is regulated by the tumor phenotype, in which the nanocarrier size needs to be considered carefully. Even more careful selection of nanocarrier size and other physical parameters is required for PCa theranostics due to their less permeable vasculature. Reports also demonstrated that the size of nanocarriers should not exceed 60 nm to allow for better tumor penetration with an efficient exit from the bloodstream. However, precise control of nanoparticle size formed through supramolecular self-assembly is a highly challenging task, whereas covalently conjugated polymers and macromolecules allow more control over their size.

In this review, we have discussed the current state-of-the-art of radiolabeled nanocarrier systems for PSMA-targeted imaging and treatment of PCa and have compared their physical properties, design composition, and in vivo pharmacokinetics. Several nanocarrier systems have been discussed, including iron oxide NPs, PLGA–PEG NPs, PEGs, and dendrimer-based macromolecules (Figure 1). Based on the current development of PSMA-targeted nanocarrier systems, the dendrimer and starPEG-based macromolecular nanosystems may be better candidates than supramolecular polymer NPs to provide better target-specific tumor uptake and penetration. The small molecule-based PSMA inhibitor ACUPA has demonstrated promising PSMA binding affinity with clinically approved [^68^Ga]PSMA-11, [^18^F]DCFPyL, and [^177^Lu]PSMA-617 for imaging and therapy of PCa. In contrast, PLGA–PEG based supramolecular NPs conjugated with ACUPA ligands show poor target-specific tumor uptake in preclinical models [49,50,51,57]. The poor performance of supramolecular polymer NPs may be due to their poor stability and large size, making them prone to aggregation and scavenging by the immune system (Figure 3A,D). However, water-soluble macromolecular systems with precisely optimized size and targeting ligand density may provide high targeted-specific tumor uptake, deeper tumor penetration, and longtime tumor retention for better therapeutic response. Comparative analysis of prior reports demonstrates that multivalent macromolecules, such as covalently conjugated nanoplexes, dendrimers, and polymers, may be better scaffolds than amphiphilic nanoparticle and lipid vesicle systems for improved tumor uptake and penetration. Low molecular weight-based agents radiolabeled with short half-life isotopes (^18^F, ^68^Ga) are very promising for diagnosis, as their high clearance rate prevents prolonged radiation exposure. However, in other cases small molecule-based therapeutic drugs are less suitable, as their fast pharmacokinetics may reduce the overall absorbed dose, requiring multiple high-dose treatments for optimum therapeutic response [8,29,76,77]. Thus, the nanocarrier systems with high payload and prolonged tumor retention could be much better candidates to those of the small molecule agents if several other physicochemical parameters, that potentially influence the in vivo pharmacokinetics of nanocarrier systems, are optimized carefully, as illustrated in Figure 3. The key design strategy of targeted nanocarriers aims to improve the bioavailability of drugs with targeted delivery of therapeutic payloads and should provide deep-tumor penetration with longer time retention. PSMA-targeted nanocarrier systems offer a vast arena of further optimization, potentially enabling future clinical translation of this promising technology. Nanocarrier systems with optimum size, ligand density, and surface functionality are yet to be realized, providing a challenging yet necessary arena for future investigation.

## Figures and Tables

**Table 1:** Chelators (including their respective derivatives) and their most suitable radiometal counterparts commonly used in biomedical applications [29]. (CA: Coordinating Atoms; CN: Coordination Number).

Name	Chemical Structures	CA	CN	Radiometals
DFO; desferrioxamine B	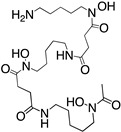	O_6_	6	^89^Zr^4+^
DTPA; diethylenetriaminepentaacetic acid	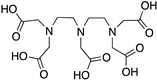	N_3_O_5_	8	^111^In^3+^, ^177^Lu^3+^, ^86/90^Y^3+^
Pa Family; H2dedpa; 1,2-[[6-(carboxy)-pyridin-2-yl]- methylamino]ethane	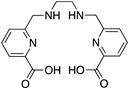	N_4_O_2_	6	^67/68^Ga^3+^,^111^In^3+^, ^177^Lu^3+^
HOPO; 3,4,3-(LI-1,2-HOPO)	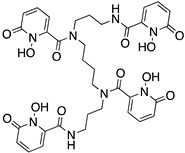	O_8_	8	^89^Zr^4+^, ^227^Th^4+^
NOTA; 1,4,7-triazacyclononane-1,4,7- triacetic acid	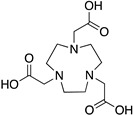	N_3_O_3_	6	^177^Lu^3+^, ^64^Cu^2+, 67/68^Ga^3+, 86/90^Y^3+, 212/213^Bi^3+^
DOTA; 1,4,7,10-tetraazacyclododecane- 1,4,7,10-tetraacetic acid, maximum	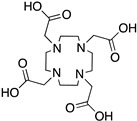	N_4_O_4_	8	^64^Cu^2+^, ^212^Pb^2+^, ^212/213^Bi^3+^, ^177^Lu^3+^, ^225^Ac^3+^, ^111^In^3+^, ^44/47^Sc^3+^, ^86/90^Y^3+^

## Data Availability

Data sharing not applicable.

## Abbreviations

ACUPA	((S)-2-(3-((S)-5-amino-1- carboxypentyl) ureido) pentanedioic acid
BNCT	boron neutron capture therapy
BSB	binding site barrier
Chk	choline kinase
DFB	deferoxamine B
DUPA	2-[3-(1,3-dicarboxypropyl)-ureido]pentanedioic acid
EPR	enhanced permeability and retention
FDA	Food and Drug Administration
GCPII	glutamate carboxypeptidase II
GRPR	gastrin-releasing peptide receptors
NPs	nanoparticles
PCa	prostate cancer
PEI	polyethyleneimine
PEG	polyethylene glycol
PET	positron emission tomography
PLA	polylactic acid
PLGA	poly(D,L-lactide-co-glycolide)
PSMA	prostate specific membrane antigen
SPECT	single-photon emission computerized tomography
TFA	trifluoroacetic acid

## References

[1] Mitchell M., Billingsley M., Haley R., Wechsler M., Peppas N., Langer R. (2021). Engineering precision nanoparticles for drug delivery. Nat. Rev. Drug Discov..

[2] Bahrami B., Hojjat-Farsangi M., Mohammadi H., Anvari E., Ghalamfarsa G., Yousefi M., Jadidi-Niaragh F. (2017). Nanoparticles and targeted drug delivery in cancer therapy. Immunol. Lett..

[3] Kratz F., Senter P., Steinhagen H. (2012). Drug Delivery in Oncology: From Basic Research to Cancer Therapy, Vols 1–3.

[4] Chauhan V., Stylianopoulos T., Martin J., Popovic Z., Chen O., Kamoun W., Bawendi M., Fukumura D., Jain R. (2012). Normalization of tumour blood vessels improves the delivery of nanomedicines in a size-dependent manner. Nat. Nanotechnol..

[5] Lim J., Guan B., Nham K., Hao G., Sun X., Simanek E. (2019). Tumor Uptake of Triazine Dendrimers Decorated with Four, Sixteen, and Sixty-Four PSMA-Targeted Ligands: Passive versus Active Tumor Targeting. Biomolecules.

[6] Goos J., Cho A., Carter L., Dilling T., Davydova M., Mandleywala K., Puttick S., Gupta A., Price W., Quinn J. (2020). Delivery of polymeric nanostars for molecular imaging and endoradiotherapy through the enhanced permeability and retention (EPR) effect. Theranostics.

[7] Heneweer C., Holland J., Divilov V., Carlin S., Lewis J. (2011). Magnitude of Enhanced Permeability and Retention Effect in Tumors with Different Phenotypes: Zr-89-Albumin as a Model System. J. Nucl. Med..

[8] Wang H., He Z., Liu X., Huang Y., Hou J., Zhang W., Ding D. (2022). Advances in Prostate-Specific Membrane Antigen (PSMA)-Targeted Phototheranostics of Prostate Cancer. Small Struct..

[9] Zhou J., Neale J., Pomper M., Kozikowski A. (2005). NAAG peptidase inhibitors and their potential for diagnosis and therapy. Nat. Rev. Drug Discov..

[10] Carter R., Feldman A., Coyle J. (1996). Prostate-specific membrane antigen is a hydrolase with substrate and pharmacologic characteristics of a neuropeptidase. Proc. Natl. Acad. Sci. USA.

[11] Davis M., Bennett M., Thomas L., Bjorkman P. (2005). Crystal structure of prostate-specific membrane antigen, a tumor marker and peptidase. Proc. Natl. Acad. Sci. USA.

[12] Filippi L., Bagni O., Nervi C. (2020). Aptamer-based technology for radionuclide targeted imaging and therapy: A promising weapon against cancer. Expert Rev. Med. Devices.

[13] Sah B., Burger I., Schibli R., Friebe M., Dinkelborg L., Graham K., Borkowski S., Bacher-Stier C., Valencia R., Srinivasan A. (2015). Dosimetry and First Clinical Evaluation of the New F-18-Radiolabeled Bombesin Analogue BAY 864367 in Patients with Prostate Cancer. J. Nucl. Med..

[14] Tsai W., Zettlitz K., Tavare R., Kobayashi N., Reiter R., Wu A. (2018). Dual-Modality ImmunoPET/Fluorescence Imaging of Prostate Cancer with an Anti-PSCA Cys-Minibody. Theranostics.

[15] Zhao N., Chopra S., Trepka K., Wang Y., Sakhamuri S., Hooshdaran N., Kim H., Zhou J., Lim S., Leung K. (2022). CUB Domain-Containing Protein 1 (CDCP1) Is a Target for Radioligand Therapy in Castration-Resistant Prostate Cancer, including PSMA Null Disease. Clin. Cancer Res..

[16] Wang S., Li J., Hua J., Su Y., Beckford-Vera D., Zhao W., Jayaraman M., Huynh T., Zhao N., Wang Y. (2021). Molecular Imaging of Prostate Cancer Targeting CD46 Using ImmunoPET. Clin. Cancer Res..

[17] Timmermand O., Elgqvist J., Beattie K., Orbom A., Larsson E., Eriksson S., Thorek D., Beattie B., Tran T., Ulmert D. (2019). Preclinical efficacy of hK2 targeted [Lu-177] hu11B6 for prostate cancer theranostics. Theranostics.

[18] Korsen J., Kalidindi T., Khitrov S., Samuels Z., Chakraborty G., Gutierrez J., Poirier J., Rudin C., Chen Y., Morris M. (2022). Molecular Imaging of Neuroendocrine Prostate Cancer by Targeting Delta-Like Ligand 3. J. Nucl. Med..

[19] Korsen J., Gutierrez J., Tully K., Carter L., Samuels Z., Khitrov S., Poirier J., Rudin C., Chen Y., Morris M. (2022). Delta-like ligand 3-targeted radioimmunotherapy for neuroendocrine prostate cancer. Proc. Natl. Acad. Sci. USA.

[20] Chou J., Egusa E.A., Wang S., Badura M.L., Lee F., Bidkar A.P., Zhu J., Shenoy T., Trepka K., Robinson T.M. (2023). Immunotherapeutic Targeting and PET Imaging of DLL3 in Small-Cell Neuroendocrine Prostate Cancer. Cancer Res..

[21] van Rij C., Frielink C., Goldenberg D., Sharkey R., Franssen G., Lutje S., McBride W., Oyen W., Boerman O. (2015). Pretargeted ImmunoPET of Prostate Cancer with an Anti-TROP-2 x Anti-HSG Bispecific Antibody in Mice with PC3 Xenografts. Mol. Imaging Biol..

[22] Filippi L., Evangelista L., Sathekge M., Schillaci O. (2022). ImmunoPET for prostate cancer in the PSMA era: Do we need other targets?. Clin. Transl. Imaging.

[23] Virgolini I., Decristoforo C., Haug A., Fanti S., Uprimny C. (2018). Current status of theranostics in prostate cancer. Eur. J. Nucl. Med. Mol. Imaging.

[24] Pastorino S., Riondato M., Uccelli L., Giovacchini G., Giovannini E., Duce V., Ciarmiello A. (2020). Toward the Discovery and Development of PSMA Targeted Inhibitors for Nuclear Medicine Applications. Curr. Radiopharm..

[25] Fendler W., Calais J., Eiber M., Flavell R., Mishoe A., Feng F., Nguyen H., Reiter R., Rettig M., Okamoto S. (2019). Assessment of Ga-68-PSMA-11 PET Accuracy in Localizing Recurrent Prostate Cancer: A Prospective Single-Arm Clinical Trial. Jama Oncol..

[26] Morris M., Rowe S., Gorin M., Saperstein L., Pouliot F., Josephson D., Wong J., Pantel A., Cho S., Gage K. (2021). Diagnostic Performance of F-18-DCFPyL-PET/CT in Men with Biochemically Recurrent Prostate Cancer: Results from the CONDOR Phase III, Multicenter Study. Clin. Cancer Res..

[27] Sartor O., de Bono J., Chi K., Fizazi K., Herrmann K., Rahbar K., Tagawa S., Nordquist L., Vaishampayan N., El-Haddad G. (2021). Lutetium-177-PSMA-617 for Metastatic Castration-Resistant Prostate Cancer. N. Engl. J. Med..

[28] Brandt M., Cardinale J., Aulsebrook M., Gasser G., Mindt T. (2018). An Overview of PET Radiochemistry, Part 2: Radiometals. J. Nucl. Med..

[29] Price E., Orvig C. (2014). Matching chelators to radiometals for radiopharmaceuticals. Chem. Soc. Rev..

[30] Crisan G., Moldovean-Cioroianu N., Timaru D., Andries G., Cainap C., Chis V. (2022). Radiopharmaceuticals for PET and SPECT Imaging: A Literature Review over the Last Decade. Int. J. Mol. Sci..

[31] (2022). FDA Approves Pluvicto/Locametz for Metastatic Castration-Resistant Prostate Cancer. J. Nucl. Med..

[32] Filippi L., Chiaravalloti A., Schillaci O., Bagni O. (2020). The potential of PSMA-targeted alpha therapy in the management of prostate cancer. Expert Rev. Anticancer Ther..

[33] Maeda H. (2015). Toward a full understanding of the EPR effect in primary and metastatic tumors as well as issues related to its heterogeneity. Adv. Drug Deliv. Rev..

[34] Matsumura Y., Maeda H. (1986). A New Concept for Macromolecular Therapeutics in Cancer-Chemotherapy—Mechanism of Tumoritropic Accumulation of Proteins and the Antitumor Agent Smancs. Cancer Res..

[35] Fang J., Nakamura H., Maeda H. (2011). The EPR effect: Unique features of tumor blood vessels for drug delivery, factors involved, and limitations and augmentation of the effect. Adv. Drug Deliv. Rev..

[36] Vakoc B., Lanning R., Tyrrell J., Padera T., Bartlett L., Stylianopoulos T., Munn L., Tearney G., Fukumura D., Jain R. (2009). Three-dimensional microscopy of the tumor microenvironment in vivo using optical frequency domain imaging. Nat. Med..

[37] Miao L., Newby J., Lin C., Zhang L., Xu F., Kim W., Forest M., Lai S., Milowsky M., Wobker S. (2016). The Binding Site Barrier Elicited by Tumor Associated Fibroblasts Interferes Disposition of Nanoparticles in Stroma-Vessel Type Tumors. Acs Nano.

[38] Regmi S., Sathianathen N., Stout T., Konety B. (2021). MRI/PET Imaging in elevated PSA and localized prostate cancer: A narrative review. Transl. Androl. Urol..

[39] Hofman M., Lawrentschuk N., Francis R., Tang C., Vela I., Thomas P., Rutherford N., Martin J., Frydenberg M., Shakher R. (2020). Prostate-specific membrane antigen PET-CT in patients with high-risk prostate cancer before curative-intent surgery or radiotherapy (proPSMA): A prospective, randomised, multicentre study. Lancet.

[40] Moon S., Yang B., Kim Y., Hong M., Lee Y., Lee D., Chung J., Jeong J. (2016). Development of a complementary PET/MR dual-modal imaging probe for targeting prostate-specific membrane antigen (PSMA). Nanomed.-Nanotechnol. Biol. Med..

[41] Liolios C., Koutsikou T., Salvanou E., Kapiris F., Machairas E., Stampolaki M., Kolocouris A., Efthimiadou E., Bouziotis P. (2022). Synthesis and in vitro proof-of-concept studies on bispecific iron oxide magnetic nanoparticles targeting PSMA and GRP receptors for PET/MR imaging of prostate cancer. Int. J. Pharm..

[42] Azad B., Banerjee S., Pullambhatla M., Lacerda S., Foss C., Wang Y., Ivkov R., Pomper M. (2015). Evaluation of a PSMA-targeted BNF nanoparticle construct. Nanoscale.

[43] Ancira-Cortez A., Ferro-Flores G., Jimenez-Mancilla N., Morales-Avila E., Trujillo-Benitez D., Ocampo-Garcia B., Santos-Cuevas C., Escudero-Castellanos A., Luna-Gutierrez M. (2020). Synthesis, chemical and biochemical characterization of Lu_2_O_3_-iPSMA nanoparticles activated by neutron irradiation. Mater. Sci. Eng. C-Mater. Biol. Appl..

[44] Hernandez-Jimenez T., Cruz-Nova P., Ancira-Cortez A., Gibbens-Bandala B., Lara-Almazan N., Ocampo-Garcia B., Santos-Cuevas C., Morales-Avila E., Ferro-Flores G. (2022). Toxicity Assessment of [Lu-177] Lu-iFAP/iPSMA Nanoparticles Prepared under GMP-Compliant Radiopharmaceutical Processes. Nanomaterials.

[45] Czerwinska M., Fracasso G., Pruszynski M., Bilewicz A., Kruszewski M., Majkowska-Pilip A., Lankoff A. (2020). Design and Evaluation of ^223^Ra-Labeled and Anti-PSMA Targeted NaA Nanozeolites for Prostate Cancer Therapy-Part I. Materials.

[46] Lankoff A., Czerwinska M., Walczak R., Karczmarczyk U., Tomczyk K., Brzoska K., Fracasso G., Garnuszek P., Mikolajczak R., Kruszewski M. (2021). Design and Evaluation of Ra-223-Labeled and Anti-PSMA Targeted NaA Nanozeolites for Prostate Cancer Therapy-Part II. Toxicity, Pharmacokinetics and Biodistribution. Int. J. Mol. Sci..

[47] Hrkach J., Von Hoff D., Ali M., Andrianova E., Auer J., Campbell T., De Witt D., Figa M., Figueiredo M., Horhota A. (2012). Preclinical Development and Clinical Translation of a PSMA-Targeted Docetaxel Nanoparticle with a Differentiated Pharmacological Profile. Sci. Transl. Med..

[48] Afsharzadeh M., Hashemi M., Babaei M., Abnous K., Ramezani M. (2020). PEG-PLA nanoparticles decorated with small-molecule PSMA ligand for targeted delivery of galbanic acid and docetaxel to prostate cancer cells. J. Cell. Physiol..

[49] Cheng J., Teply B., Sherifi I., Sung J., Luther G., Gu F., Levy-Nissenbaum E., Radovic-Moreno A., Langer R., Farokhzad O. (2007). Formulation of functionalized PLGA-PEG nanoparticles for in vivo targeted drug delivery. Biomaterials.

[50] Gu F., Zhang L., Teply B., Mann N., Wang A., Radovic-Moreno A., Langer R., Farokhzad O. (2008). Precise engineering of targeted nanoparticles by using self-assembled biointegrated block copolymers. Proc. Natl. Acad. Sci. USA.

[51] Banerjee S., Foss C., Horhota A., Pullambhatla M., McDonnell K., Zale S., Pomper M. (2017). In-111- and IRDye800CW-Labeled PLA-PEG Nanoparticle for Imaging Prostate-Specific Membrane Antigen-Expressing Tissues. Biomacromolecules.

[52] Hu K., Yang Z., Zhang L., Xie L., Wang L., Xu H., Josephson L., Liang S., Zhang M. (2020). Boron agents for neutron capture therapy. Coord. Chem. Rev..

[53] Barth R., Mi P., Yang W. (2018). Boron delivery agents for neutron capture therapy of cancer. Cancer Commun..

[54] Xuan S., Vicente M.d.G.H. (2018). Recent Advances in Boron Delivery Agents for Boron Neutron Capture Therapy (BNCT). Boron-Based Compounds.

[55] Haapaniemi A., Kankaanranta L., Saat R., Koivunoro H., Saarilahti K., Makitie A., Atula T., Joensuu H. (2016). Boron Neutron Capture Therapy in the Treatment of Recurrent Laryngeal Cancer. Int. J. Radiat. Oncol. Biol. Phys..

[56] Wang S., Blaha C., Santos R., Huynh T., Hayes T., Beckford-Vera D., Blecha J., Hong A., Fogarty M., Hope T. (2019). Synthesis and Initial Biological Evaluation of Boron-Containing Prostate-Specific Membrane Antigen Ligands for Treatment of Prostate Cancer Using Boron Neutron Capture Therapy. Mol. Pharm..

[57] Meher N., Seo K., Wang S., Bidkar A., Fogarty M., Dhrona S., Huang X., Tang R., Blaha C., Evans M. (2021). Synthesis and Preliminary Biological Assessment of Carborane-Loaded Theranostic Nanoparticles to Target Prostate-Specific Membrane Antigen. Acs Appl. Mater. Interfaces.

[58] Vera D., Fontaine S., VanBrocklin H., Hearn B., Reid R., Ashley G., Santi D. (2020). PET Imaging of the EPR Effect in Tumor Xenografts Using Small 15 nm Diameter Polyethylene Glycols Labeled with Zirconium-89. Mol. Cancer Ther..

[59] Chen Z., Tai Z., Gu F., Hu C., Zhu Q., Gao S. (2016). Aptamer-mediated delivery of docetaxel to prostate cancer through polymeric nanoparticles for enhancement of antitumor efficacy. Eur. J. Pharm. Biopharm..

[60] Sercombe L., Veerati T., Moheimani F., Wu S., Sood A., Hua S. (2015). Advances and Challenges of Liposome Assisted Drug Delivery. Front. Pharmacol..

[61] Cheng M., Overchuk M., Rajora M., Lou J., Chen Y., Pomper M., Chen J., Zheng G. (2022). Targeted Theranostic 111In/Lu-Nanotexaphyrin for SPECT Imaging and Photodynamic Therapy. Mol. Pharm..

[62] Vaughan L., Glanzel W., Korch C., Capes-Davis A. (2017). Widespread Use of Misidentified Cell Line KB (HeLa): Incorrect Attribution and Its Impact Revealed through Mining the Scientific Literature. Cancer Res..

[63] Zhu C., Bandekar A., Sempkowski M., Banerjee S., Pomper M., Bruchertseifer F., Morgenstern A., Sofou S. (2016). Nanoconjugation of PSMA-Targeting Ligands Enhances Perinuclear Localization and Improves Efficacy of Delivered Alpha-Particle Emitters against Tumor Endothelial Analogues. Mol. Cancer Ther..

[64] Wong P., Li L., Chea J., Delgado M., Crow D., Poku E., Szpikowska B., Bowles N., Channappa D., Colcher D. (2017). PET imaging of Cu-64-DOTA-scFv-anti-PSMA lipid nanoparticles (LNPs): Enhanced tumor targeting over anti-PSMA scFv or untargeted LNPs. Nucl. Med. Biol..

[65] Wong P., Li L., Chea J., Delgado M., Poku E., Szpikowska B., Bowles N., Minnix M., Colcher D., Wong J. (2017). Synthesis, Positron Emission Tomography Imaging, and Therapy of Diabody Targeted Drug Lipid Nanoparticles in a Prostate Cancer Murine Model. Cancer Biother. Radiopharm..

[66] Davis M., Zuckerman J., Choi C., Seligson D., Tolcher A., Alabi C., Yen Y., Heidel J., Ribas A. (2010). Evidence of RNAi in humans from systemically administered siRNA via targeted nanoparticles. Nature.

[67] Chen Z., Penet M., Nimmagadda S., Li C., Banerjee S., Winnard P., Artemov D., Glunde K., Pomper M., Bhujwalla Z. (2012). PSMA-Targeted Theranostic Nanoplex for Prostate Cancer Therapy. Acs Nano.

[68] Lesniak W., Boinapally S., Banerjee S., Azad B., Foss C., Shen C., Lisok A., Wharram B., Nimmagadda S., Pomper M. (2019). Evaluation of PSMA-Targeted PAMAM Dendrimer Nanoparticles in a Murine Model of Prostate Cancer. Mol. Pharm..

[69] Meher N., Ashley G., Bidkar A., Dhrona S., Fong C., Fontaine S., Vera D., Wilson D., Seo Y., Santi D. (2022). Prostate-Specific Membrane Antigen Targeted Deep Tumor Penetration of Polymer Nanocarriers. Acs Appl. Mater. Interfaces.

[70] Gratton S., Ropp P., Pohlhaus P., Luft J., Madden V., Napier M., DeSimone J. (2008). The effect of particle design on cellular internalization pathways. Proc. Natl. Acad. Sci. USA.

[71] Safari H., Kelley W., Saito E., Kaczorowski N., Carethers L., Shea L., Eniola-Adefeso O. (2020). Neutrophils preferentially phagocytose elongated particles-An opportunity for selective targeting in acute inflammatory diseases. Sci. Adv..

[72] Elci S., Jiang Y., Yan B., Kim S., Saha K., Moyano D., Tonga G., Jackson L., Rotello V., Vachet R. (2016). Surface Charge Controls the Suborgan Biodistributions of Gold Nanoparticles. Acs Nano.

[73] Pijeira M., Viltres H., Kozempel J., Sakmar M., Vlk M., Ilem-Ozdemir D., Ekinci M., Srinivasan S., Rajabzadeh A., Ricci-Junior E. (2022). Radiolabeled nanomaterials for biomedical applications: Radiopharmacy in the era of nanotechnology. Ejnmmi Radiopharm. Chem..

[74] Androvic L., Woldrichova L., Jozefjakova K., Pechar M., Lynn G., Kankova D., Malinova L., Laga R. (2021). Cyclotriphosphazene-Based Star Copolymers as Structurally Tunable Nanocarriers with Programmable Biodegradability. Macromolecules.

[75] Ernsting M., Murakami M., Roy A., Li S. (2013). Factors controlling the pharmacokinetics, biodistribution and intratumoral penetration of nanoparticles. J. Control. Release.

[76] Hofmann M., Maecke H., Borner A., Weckesser E., Schoffski P., Oei M., Schumacher J., Henze M., Heppeler A., Meyer G. (2001). Biokinetics and imaging with the somatostatin receptor PET radioligand Ga-68-DOTATOC: Preliminary data. Eur. J. Nucl. Med..

[77] Debela D., Muzazu S., Heraro K., Ndalama M., Mesele B., Haile D., Kitui S., Manyazewal T. (2021). New approaches and procedures for cancer treatment: Current perspectives. Sage Open Med..

